# Associations of carbohydrate quality and cardiovascular risk factors vary among diabetes subtypes

**DOI:** 10.1186/s12933-025-02580-4

**Published:** 2025-02-06

**Authors:** Katharina S. Weber, Sabrina Schlesinger, Janina Goletzke, Klaus Straßburger, Oana-Patricia Zaharia, Sandra Trenkamp, Robert Wagner, Wolfgang Lieb, Anette E. Buyken, Michael Roden, Christian Herder, for the GDS group, M. Roden, H. Al-Hasani, B. Belgardt, G. Bönhof, G. Geerling, R. Guthoff, C. Herder, A. Icks, K. Jandeleit-Dahm, J. Kotzka, O. Kuß, E. Lammert, W. Rathmann, S. Schlesinger, V. Schrauwen-Hinderling, J. Szendroedi, S. Trenkamp, R. Wagner

**Affiliations:** 1 Institute of Epidemiology, Kiel University, Niemannsweg 11, 24105 Kiel, Germany; 2 Institute for Biometrics and Epidemiology, German Diabetes Center, Leibniz Center for Diabetes Research at Heinrich Heine University Düsseldorf, Düsseldorf, Germany; 3 German Center for Diabetes Research (DZD), Partner Düsseldorf, Munich-Neuherberg, Germany; 4 Faculty of Natural Sciences, Institute of Nutrition, Consumption and Health, Paderborn University, Paderborn, Germany; 5 Department of Endocrinology and Diabetology, Medical Faculty and University Hospital Düsseldorf, Heinrich Heine University Düsseldorf, Düsseldorf, Germany; 6 Institute for Clinical Diabetology, German Diabetes Center, Leibniz Center for Diabetes Research at Heinrich Heine University Düsseldorf, Düsseldorf, Germany

**Keywords:** Glycemic index, Glycemic load, Diabetes clusters, Diabetes-related complications

## Abstract

**Background:**

Assess the intake of carbohydrate quality and their association with cardiovascular risk factors among diabetes subtypes.

**Methods:**

Participants of the German Diabetes Study (GDS) (recent-onset diabetes (n = 487) and 5-years thereafter (n = 209)) were allocated into severe autoimmune diabetes (SAID, 35%), severe insulin-deficient diabetes (SIDD, 3%), severe insulin-resistant diabetes (SIRD, 5%), mild obesity-related diabetes (MOD, 28%), and mild age-related diabetes (MARD, 29%). Dietary glycemic index (GI), glycemic load (GL), and intake of higher- (≥ 55) and low-GI (< 55) foods, dietary fiber, and total sugar were derived from a validated food frequency questionnaire and cross-sectionally associated with cardiovascular risk factors (blood lipids, subclinical inflammation, blood pressure, fatty liver index) using multivariable linear regression analysis for subtypes with prevalences ≥ 10%.

**Results:**

Intake of carbohydrate quality parameters was broadly comparable between the subtypes. Among SAID higher total sugar intake was associated with lower HDL-cholesterol (ß (95% CI) relative change per 1 SD increment: − 3.4% (− 6.7; − 0.1)). No clear associations were seen among MOD. Among MARD, a higher dietary GL and higher-GI carbohydrate intake were associated with higher serum triglycerides (10.9% (2.4; 20.1), 12.4% (3.9; 21.5)) and fatty liver index (absolute change: 0.18 (0.06; 0.31), 0.17 (0.05; 0.28)) and lower HDL-cholesterol (− 4.1% (− 7.6; − 0.4), − 4.4% (− 7.8; − 0.8)), whilst higher intake of low-GI carbohydrates and dietary fiber were associated with lower high-sensitivity C-reactive protein (− 16.0% (− 25.7; − 5.1), − 13.9% (− 24.2; − 2.2)).

**Conclusions:**

Associations of carbohydrate quality parameters with blood lipids, subclinical inflammation, and fatty liver index differed between diabetes subtypes. However, evidence is too preliminary to derive subtype-specific recommendations.

**Trial registration:**

Clinicaltrials.gov: NCT01055093.

**Supplementary Information:**

The online version contains supplementary material available at 10.1186/s12933-025-02580-4.

## Background

Diabetes mellitus comprises a group of metabolic disorders characterized by hyperglycemia [1]. The increased risk of diabetes-related complications, such as cardiovascular disease, should be addressed as part of a multifactorial approach in which diet is a crucial aspect [2]. We have previously shown that the five diabetes subtypes according to the reclassification approach by Ahlqvist et al. [3, 4] showed only minor differences in adherence to healthy dietary patterns, but nevertheless differed in their associations of dietary pattern adherence with cardiovascular risk factors, ie. blood lipids, subclinical inflammation, blood pressure, and liver fat content [5]. In addition, these subtypes, which comprise severe autoimmune diabetes (SAID), severe insulin-deficient diabetes (SIDD), severe insulin-resistant diabetes (SIRD), mild obesity-related diabetes (MOD), and mild age-related diabetes (MARD), show distinct disease progression and differ in their risk of diabetes-related complications [3, 4, 6]. Thus, it is important to investigate carbohydrate quality as a further dietary characteristic in relation to the subtypes and their risk of cardiovascular disease.

Carbohydrate quality plays an important role in the treatment of people with diabetes [7, 8] as the postprandial glycemic response is primarily affected by carbohydrates [9]. Carbohydrate quality can be captured by e. g., dietary fiber, whole grain content, and dietary sugar [10, 11]. The addition of the glycemic index (GI) also enables the consideration of carbohydrate-induced postprandial glycemic excursions as the GI classifies carbohydrate-rich foods according to their relative impact on blood glucose responses [10, 12]. The glycemic load (GL) considers both, the GI of the foods and the amount of carbohydrate eaten as it is calculated as the product of GI and the total available carbohydrate content in a serving [2, 12]. According to current dietary guidelines for people with diabetes, carbohydrate quality comes before quantity and low-GI (GI ≤ 55) or low-GL diets are preferable, if their composition is consistent with e. g., recommendations for dietary fiber and sugars [13, 14]. Lowering the GI or the GL of the diet has been shown to improve cardiovascular risk factors such as the blood lipid profile primarily among people with type 2 diabetes [15].

However, as all research on the associations of quality of carbohydrate intake with cardiovascular risk factors has been conducted categorizing people with diabetes classically as type 1 and type 2 diabetes yet, the main objectives of this cross-sectional study were to investigate (i) whether diabetes subtypes differ in their quality of carbohydrate intake and (ii) whether associations of quality of carbohydrate intake with cardiovascular risk factors are specific for the subtypes. 

## Methods

### Study population

Participants of the German Diabetes Study (GDS), an ongoing observational cohort [6, 16], were recruited consecutively from the study center Düsseldorf between 08/2012 and 06/2023. They had to fulfill the following criteria: (i) diabetes diagnosis according to American Diabetes Association criteria [1], (ii) known diabetes duration of < 12 months (baseline examination) or participation in the 5-year follow-up examination, (iii) complete data from the food frequency questionnaire (FFQ) and the GI-extended FFQ, which were both implemented in the GDS in 08/2012. If people provided dietary data from both examination time points, they were included in the cross-sectional analysis with their baseline data. Individuals with missing data for variables necessary for allocation to diabetes subtypes, outcome variables, or potential confounding factors were excluded from analysis (Suppl. Figure 1). Of note, due to identical examinations at both baseline and follow-up, excellent comparability was ensured across examinations [5, 6, 16]. The study protocol has been approved by the ethics board of Heinrich Heine University (ref. 4508) and the GDS is conducted according to the ethical standards as laid down in the 1964 Declaration of Helsinki. All participants gave their written informed consent.

### Assessment of quality of carbohydrate intake

As described before, dietary information was obtained from the validated semi-quantitative FFQ of the European Prospective Investigation into Cancer and Nutrition (EPIC)-Potsdam study, which assessed habitual food consumption frequencies of 148 food items during the past 12 months considering average portion size [5, 16, 17]. This established FFQ was extended by GI-specific questions. The GI-extended food-frequency questionnaire has been developed and validated for people with diabetes within the GDS with the aim to more accurately estimate the dietary GI in people with diabetes [18]. In brief, carbohydrate-containing food groups known to vary substantially in the GI of their individual foods were identified from the EPIC-FFQ, ie. breakfast cereals, bread and buns, pasta and rice, potatoes and potato products, juices and lemonades, savory snacks, and cakes. Of these, the food items consumed most frequently among German adults were listed within the GI-extension questionnaire using the same consumption frequency categories as for the respective food of the EPIC-FFQ. Published GI values were then assigned to both, the carbohydrate-containing food items of the GI-extension as well as the EPIC-FFQ itself. Finally, the overall dietary GI and GL were estimated for each individual by combining the information from both questionnaires. For analysis, carbohydrate-containing food items were differentiated into higher-GI (≥ 55, ie. moderate to high-GI) and low-GI (< 55) foods [11, 18]. Carbohydrate quality was also characterized by dietary fiber intake data obtained from the EPIC-FFQ and total sugar intake. Total sugar intake was defined as the sum of mono- and disaccharides [19], supplemented by information from the GI-extension questionnaire since the EPIC-FFQ version used in the GDS does not distinguish between sugar-sweetened and non-sugar-sweetened beverages [5, 18]. Thus, also for total daily energy intake, the EPIC-FFQ data were supplemented by information from the GI-extension questionnaire.

Of note, misreported FFQ data according to the cutoffs for implausible energy intake (ie. men < 800 kcal and > 4000 kcal, women < 500 kcal and > 3500 kcal, respectively) as defined by Walter Willett [20] was evident for 14 individuals (2%) who overreported their total daily energy intake.

### Procedures

*Variables necessary for allocation to diabetes subtypes (clustering variables).* Assessment of the clustering variables has been described before [5, 6, 16]. Briefly, glutamic decarboxylase antibodies (GADA) were quantified by radioligand assay and HbA1c was measured on a Variant-II (Bio-Rad, Munich, Germany). Using the HOMA calculator [21] (University of Oxford, Oxford, UK), homoeostasis model assessment of β-cell function (HOMA2-B) and insulin resistance (HOMA2-IR) were calculated based on fasting glucose and C-peptide concentrations. Body mass index (BMI) was calculated as body weight divided by the square of body height [5, 6, 16].

*Outcome variables.* Cardiovascular risk factors, ie. serum lipid concentrations, high sensitivity C-reactive protein (hsCRP), blood pressure, and the fatty liver index (FLI), were chosen as outcome variables for this analysis. Serum concentrations of triglycerides as well as total, high-density lipoprotein (HDL), and low-density lipoprotein (LDL) cholesterol were measured on a Cobas c311 (Roche Diagnostics, Mannheim, Germany) [16]. hsCRP was quantified on a Roche/Hitachi c 311 analyzer (Basel, Switzerland) [22]. Systolic and diastolic blood pressure were measured three times in the right arm while sitting using a validated automatic device (OMRON 705IT, OMRON HEALTHCARE, Germany) and taking the mean of the second and third measurement for analysis [23]. We used the FLI (expressed in arbitrary units) as a validated surrogate of liver fat content, which was derived from concentrations of triglycerides and gamma-glutamyl transpeptidase, BMI, and waist circumference as described before [24].

*Covariates.* Using standardized questionnaires, information on age, sex, current smoking status (yes/no), glucose-lowering medication (insulin (with or without oral glucose-lowering drugs)/metformin/metformin and oral glucose-lowering drugs/other/none), antihypertensive medication (yes/no), and lipid-lowering medication (yes/no) was obtained [16]. Socioeconomic status was defined as a multidimensional aggregated score based on income, education, and occupation, which were all assessed by standardized questionnaires [25]. In addition, the partnership status (with/without spouse or partner) was obtained by questionnaire. A physical activity index covering leisure and sport activities, was derived as a modified version of the Baecke Index [26, 27].

### Statistical analyses

All statistical analyses were conducted using SAS® version 9.4 (SAS Institute, Cary, NC, USA).

*Diabetes subtype allocation.* According to the sex-specific classification rules as published by Ahlqvist et al. [3] and repeatedly applied to the GDS [5, 6, 28–32], people with diabetes were allocated into one of the five predefined subtypes (clusters), ie. SAID, SIDD, SIRD, MOD, and MARD based on six variables, ie. age at diagnosis, BMI, HbA1c, HOMA2-B, HOMA2-IR, and GADA. Individuals with positive GADA were assigned to the SAID cluster, while the remaining individuals were assigned to the other four clusters using the nearest centroid approach. The clustering tool is available online at https://diabetescalculator.ddz.de. Importantly, diabetes subtype allocation was made at the respective examination time point [5].

*Comparison of quality of carbohydrate intake between subtypes.* Overall differences in quality of carbohydrate intake between the diabetes subtypes were analyzed using the Kruskal–Wallis test.

*Associations of quality of carbohydrate intake with outcome variables and differences between diabetes subtypes.* Multivariable linear regression analysis adjusted for potential confounders was used to assess associations of quality of carbohydrate intake (continuous independent variable) with cardiovascular risk factors (each continuous variable considered separate as dependent variable). Confounders were selected a-priori based on the literature. Dietary GL, low-GI and higher-GI carbohydrates, dietary fiber, and total sugar intake were energy adjusted using the residual method. As the residuals of models with the outcomes hsCRP and serum lipid concentrations were not linear, these variables were entered into the models as ln-transformed variables and back-transformed for data presentation. Models were adjusted for age [years], sex [male/female], BMI [kg/m^2^], current smoking status [current non-smoking/smoking], total daily energy intake [kcal/d], socioeconomic index [continuous], partnership status [with/without spouse or partner], and physical activity index [continuous]. Models with serum lipid concentrations as dependent variable were additionally adjusted for glucose-lowering medication [insulin (with or without oral glucose-lowering drugs)/metformin/metformin and oral glucose-lowering drugs/other/none] and lipid-lowering medication [yes/no], models with hsCRP as dependent variable additionally considered glucose-lowering medication as potential confounder, and models including blood pressure as dependent variable were additionally adjusted for antihypertensive medication [yes/no]. Models with the FLI as dependent variable were additionally adjusted for total daily alcohol intake [g/d] and glucose- and lipid-lowering medication.

Interaction analyses were applied to test for differences between subtypes in their association of quality of carbohydrate intake with the respective outcome variable by adding multiplicative interaction terms (parameters of carbohydrate quality*diabetes subtypes). Due to the low absolute number of individuals with SIDD and SIRD, these two subtypes were excluded from association and interaction analyses.

*Sensitivity analyses.* Association and interaction analyses were repeated including only individuals with recently diagnosed diabetes from the baseline examination in order to test the reproducibility of the results in a more homogeneous cohort. In a further sensitivity analysis, the final model was additionally adjusted for total fat and total protein intake (energy adjusted using the residual method) in order to assess the relevance of the concomitant macronutrients for the association of carbohydrate quality with cardiovascular risk factors.

## Results

*Participants’ characteristics.* We included a total of 696 individuals with diabetes (487 newly diagnosed at baseline and 209 from the 5-year follow-up examination) in our cross-sectional study (Suppl. Figure 1). Diabetes subtype allocation was as follows: 246 (35%) were assigned to the **SAID** subtype, 19 (3%) were assigned to the **SIDD**, 37 (5%) to the **SIRD**, 194 (28%) to the **MOD**, and 200 (29%) to the **MARD** subtype (Table 1). Table 1 presents the clinical characteristics of the study population, stratified by diabetes subtype. The dietary characteristics are presented in Table 2. Median carbohydrate intake of the five subtypes ranged between 36 EN% and 39 EN% with a median total daily energy intake of 1995 kcal of the total study sample. The main sources of carbohydrate intake were comparable between subtypes with cereal (products) providing the most carbohydrates, followed by fruit or sugar and confectionary (Table 2).

**Table 1:** Clinical characteristics of the study population according to diabetes subtype allocation

	Total	SAID	SIDD	SIRD	MOD	MARD
*N (% of study sample)*^*a*^	*696 (100)*	*246 (35.3)*	*19 (2.7)*	*37 (5.3)*	*194 (27.9)*	*200 (28.7)*
Age, years^b^	48.4 ± 12.9	39.4 ± 11.6	44.1 ± 11.7	59.7 ± 8.8	47.4 ± 9.4	58.8 ± 8.1
Age at diabetes diagnosis, years^b^	46.9 ± 12.7	37.9 ± 11.6	42.5 ± 11.5	58.2 ± 7.9	45.8 ± 9.4	57.3 ± 7.2
Sex (men), n (%)^a^	429 (61.6)	130 (52.9)	15 (79.0)	29 (78.4)	103 (53.1)	152 (76.0)
*Participation time point, n (%)*^*a*^						
Baseline	487 (70.0)	176 (71.5)	13 (68.4)	26 (70.3)	133 (68.6)	139 (69.5)
5-year follow-up	209 (30.0)	70 (28.5)	6 (31.6)	11 (29.7)	61 (31.4)	61 (30.5)
***Anthropometric and clinical characteristics***						
BMI, kg/m^2b^	29.3 ± 6.4	25.6 ± 4.9	27.7 ± 3.3	35.6 ± 4.8	35.2 ± 5.9	27.3 ± 3.5
Waist circumference, cm^b^	99.1 ± 17.1	87.4 ± 14.1	97.4 ± 8.6	119.8 ± 11.4	112.3 ± 14.5	97.1 ± 10.9
Waist-to-hip ratio^b^	0.93 ± 0.09	0.87 ± 0.09	0.94 ± 0.06	1.01 ± 0.09	0.96 ± 0.08	0.95 ± 0.07
Fasting plasma glucose, mmol/L^c^	7.2 (6.2; 8.5)	7.3 (6.2; 9.2)	11.2 (9.0; 13.8)	6.9 (5.9; 8.0)	7.3 (6.4; 8.6)	6.9 (6.1; 7.9)
Fasting plasma glucose, mg/dL^c^	130 (112; 154)	132 (111; 165)	202 (162; 249)	124 (107; 145)	132 (116; 155)	125 (110; 142.5)
Fasting plasma C-peptide, nmol/L^c^	0.7 (0.3; 1.1)	0.2 (0.1; 0.4)	0.6 (0.4; 0.9)	1.9 (1.6; 2.2)	1.1 (0.9; 1.4)	0.8 (0.5; 1.0)
Fasting plasma C-peptide, ng/mL^c^	2.2 (0.9; 3.4)	0.7 (0.3; 1.2)	1.9 (1.3; 2.6)	5.8 (4.7; 6.6)	3.3 (2.6; 4.2)	2.3 (1.6; 2.9)
HOMA2-IR^c^	1.8 (0.8; 2.9)	0.6 (0.3; 1.0)	1.8 (1.2; 2.9)	4.8 (3.9; 5.8)	2.8 (2.1; 3.5)	1.9 (1.3; 2.5)
HOMA2-B, %^c^	62.9 (35.3; 95.7)	32.5 (12.7; 48.9)	27.6 (22.7; 45.5)	149.2 (124.7; 172.2)	86.0 (62.9; 118.4)	72.8 (53.3; 94.8)
HbA1c, %^c^	6.4 (6.0; 7.1)	6.6 (6.1; 7.1)	8.8 (8.0; 9.2)	6.2 (5.9; 6.7)	6.3 (5.9; 7.1)	6.3 (5.9; 6.7)
HbA1c, mmol/mol^c^	46.4 (42.1; 54.1)	48.6 (43.1; 54.1)	72.7 (63.9; 77.0)	44.2 (41.0; 49.7)	45.3 (41.0; 54.1)	45.3 (41.0; 49.7)
Participants with HbA1c < 7%, n (%)^a^	499 (71.7)	167 (33.5)	0 (0.0)	29 (5.8)	137 (27.5)	166 (33.3)
Participants with HbA1c ≥ 7%, n (%)^a^	197 (28.3)	79 (40.1)	19 (9.6)	8 (4.1)	57 (28.9)	34 (17.3)
Known diabetes duration, months^b^	24.3 ± 28.5	23.8 ± 28.2	24.1 ± 29.4	23.2 ± 29.1	25.2 ± 29.0	24.3 ± 28.5
GADA positivity at baseline, n (%)^a^	246 (35.3)	246 (100)	0 (0)	0 (0)	0 (0)	0 (0)
***Cardiovascular risk factors***						
*N (% of study sample)*^*a*^	*694 (100)*	*245 (35.3)*	*19 (2.7)*	*37 (5.3)*	*193 (27.8)*	*200 (28.8)*
*Glucose-lowering medication, n (%)*^*a*^						
Insulin (+ OAD)	236 (34.0)	178 (72.7)	4 (21.1)	2 (5.4)	28 (14.5)	24 (12.0)
Metformin	62 (8.9)	8 (3.3)	3 (15.8)	10 (27.0)	21 (10.9)	20 (10.0)
Metformin + OAD	10 (1.4)	1 (0.4)	1 (5.3)	2 (5.4)	3 (1.6)	3 (1.5)
Other	33 (4.8)	0 (0.0)	0 (0.0)	3 (8.1)	17 (8.8)	13 (6.5)
None	353 (50.9)	58 (23.7)	11 (57.9)	20 (54.1)	124 (64.3)	140 (70.0)
Antihypertensive medication (yes), n (%)^a^	244 (35.2)	25 (10.2)	9 (47.4)	26 (70.3)	91 (47.2)	93 (46.5)
Lipid-lowering medication (yes), n (%)^a^	89 (12.8)	11 (4.5)	3 (15.8)	14 (37.8)	22 (11.4)	39 (19.5)
hsCRP, mg/dL^c^	0.16 (0.07; 0.37)	0.09 (0.05; 0.22)	0.28 (0.14; 0.41)	0.34 (0.18; 0.64)	0.32 (0.14; 0.6)	0.15 (0.07; 0.28)
Diastolic blood pressure, mmHg^b^	83.3 ± 10.4	79.4 ± 9.2	87.5 ± 9.6	86.6 ± 12.1	86.9 ± 10.5	83.7 ± 10.0
Systolic blood pressure, mmHg^b^	136.6 ± 17.0	128.8 ± 14.2	143.9 ± 14.5	143.8 ± 14.8	138.5 ± 15.7	142.2 ± 18.4
Total cholesterol, mg/dL^c^	193 (165; 219)	193 (165; 219)	183 (159; 208)	215 (170; 244)	190 (175; 211)	196 (171; 226)
High-density lipoprotein cholesterol, mg/dL^c^	50 (40; 63)	50 (40; 63)	63 (49; 76)	46 (38; 56)	43 (35; 50)	44 (37; 53)
Low-density lipoprotein cholesterol, mg/dL^c^	121 (97; 146)	121 (97; 146)	107 (88; 131)	138 (110; 173)	118 (101; 138)	129 (107; 152)
Triglycerides, mg/dL^c^	109 (75; 172)	109 (75; 172)	78 (59; 109)	149 (83; 205)	217 (132; 253)	138 (101; 201)
Fatty liver index (FLI)^c^	61.8 (20.3; 89.5)	17.4 (6.5; 40.3)	67.5 (31.5; 85.5)	96.8 (92.2; 98.5)	92.2 (77.6; 97.3)	61.0 (34.7; 76.8)
*Fatty liver index (FLI), n (%)*^*a*^						
Ruling out fatty liver	229 (33.0)	168 (68.6)	3 (15.8)	0 (0.0)	15 (7.8)	43 (21.5)
Intermediate	108 (15.6)	40 (16.3)	5 (26.3)	1 (2.7)	9 (4.7)	53 (26.5)
Ruling in fatty liver	357 (51.4)	37 (15.1)	11 (57.9)	36 (97.3)	169 (87.6)	104 (52.0)
***Further covariates***						
Physical activity index (leisure and sport activities)^b^	5.9 ± 1.3	6.2 ± 1.4	5.6 ± 1.4	5.2 ± 1.1	5.4 ± 1.1	6.0 ± 1.3
Current smoking status (yes), n (%)^a^	154 (22.1)	55 (22.4)	9 (47.4)	10 (27.0)	40 (20.6)	40 (20.0)
Partnership status (with spouse or partner), n (%)^a^	517 (74.3)	177 (72.0)	13 (68.4)	28 (75.7)	135 (69.6)	164 (82.0)
*Socioeconomic status, n (%)*^*a*^						
Lower	37 (5.3)	16 (6.5)	2 (10.5)	1 (2.7)	12 (6.2)	6 (3.0)
Middle	354 (50.9)	119 (48.4)	13 (68.4)	25 (67.6)	106 (54.9)	91 (45.5)
Upper	305 (43.8)	111 (45.1)	4 (21.1)	11 (29.7)	76 (39.2)	103 (51.5)

Data are presented as ^a^n (%), ^b^mean ± standard deviation, or ^c^median (25th percentile; 75th percentile)

GADA, glutamic acid decarboxylase antibody; MARD, moderate age-related diabetes; MOD, moderate obesity-related diabetes; OAD, oral glucose-lowering drugs; SAID, severe autoimmune diabetes; SIDD, severe insulin-deficient diabetes; SIRD, severe insulin-resistant diabetes

**Table 2:** Dietary characteristics of the study population according to diabetes subtype allocation

	Total	SAID	SIDD	SIRD	MOD	MARD
*N (% of study sample)*^*a*^	*696 (100)*	*246 (35.3)*	*19 (2.7)*	*37 (5.3)*	*194 (27.9)*	*200 (28.7)*
Dietary GI^b^	55.8 ± 3.2	56.0 ± 3.3	56.2 ± 2.4	55.9 ± 2.7	56.2 ± 3.1	55.3 ± 3.3
Dietary GL^c^	103 (82; 135)	109 (83; 141)	81 (73; 149)	91 (827; 135)	104 (83; 131)	99 (76; 129)
GL/1000 kcal^b^	52.9 ± 9.9	52.5 ± 10.4	54.8 ± 8.7	52.0 ± 8.6	53.6 ± 8.4	52.5 ± 10.8
Low-GI carbohydrates, gram^c,d^	76.8 (58.9; 98.9)	79.0 (61.4; 102.9)	63.2 (42.0; 88.9)	71.4 (55.9; 91.1)	75.1 (56.3; 95.7)	76.0 (59.2; 99.3)
Low-GI carbohydrates, EN%^c,d^	16 (12; 19)	15 (12; 18)	13 (11; 19)	15 (11; 17)	16 (13; 19)	16 (12; 19)
Higher-GI carbohydrates, gram^c,e^	100.3 (76.6; 149.6)	104.6 (79.6; 163.0)	98.2 (75.2; 172.6)	99.0 (78.1; 162.6)	103.8 (78.7; 139.3)	94.7 (73.1; 142.7)
Higher-GI carbohydrates, EN%^c,e^	21 (17; 26)	21 (17; 26)	25 (19; 28)	21 (20; 24)	22 (18; 26)	21 (17; 26)
Dietary fiber, gram^c^	22.4 (17.5; 28.4)	23.4 (17.9; 29.8)	17.1 (13.8; 26.3)	21.7 (18.3; 26.3)	21.7 (16.9; 27.9)	21.9 (17.6; 27.3)
Dietary fiber/1000 kcal^c^	10.0 (8.5; 11.7)	10.0 (8.5; 11.5)	9.2 (8.3; 10.3)	9.5 (8.2; 11.4)	10.0 (8.4; 11.8)	10.4 (8.8; 12.1)
Total sugar, gram^c^	83.4 (66.3; 107.9)	87.4 (68.6; 109.7)	68.8 (51.3; 107.3)	80.1 (65.3; 93.6)	83.6 (64.3; 108.2)	81.9 (66.2; 102.1)
Total sugar, EN%^c^	17 (14; 20)	17 (14; 19)	16 (14; 21)	16 (14; 20)	18 (14; 21)	17 (14; 21)
Total daily energy intake, kcal^c^	1995 (1616; 2474)	2063 (1707; 2597)	1730 (1320; 2260)	2048 (1605; 2376)	1936 (1572; 2437)	1926 (1608; 2393)
Carbohydrates, EN%^c^	38 (34; 41)	38 (34; 41)	39 (35; 42)	36 (34; 38)	38 (35; 41)	38 (34; 42)
*Main sources of carbohydrate intake, EN%*^*c*^						
Cereal (products)	13.8 (11.0; 18.0)	14.1 (10.9; 18.7)	15.4 (12.4; 18.7)	12.9 (10.4; 16.9)	14.0 (11.4; 17.7)	12.8 (10.4; 17.4)
Fruit	4.3 (2.7; 6.8)	4.4 (2.8; 6.7)	3.1 (2.0; 5.6)	4.2 (2.8; 5.9)	4.2 (2.8; 6.2)	4.5 (2.5; 7.5)
Sugar and confectionary	3.4 (2.3; 5.0)	3.5 (2.5; 5.2)	3.6 (2.8; 4.2)	2.7 (2.4; 4.5)	3.6 (2.4; 5.3)	3.2 (2.0; 5.0)
Dairy products	2.6 (1.8; 4.0)	2.6 (1.7; 3.9)	2.7 (2.1; 3.7)	2.4 (1.8; 3.5)	2.6 (1.8; 4.2)	2.8 (1.8; 4.1)
Cake	2.5 (1.6; 3.8)	2.5 (1.7; 3.9)	2.5 (1.4; 4.1)	2.4 (1.6; 4.1)	2.6 (1.7; 4.0)	2.3 (1.5; 3.5)
Potatoes and other tubers	1.5 (0.9; 2.4)	1.4 (0.9; 2.3)	1.4 (1.1; 1.9)	2.0 (1.3; 3.0)	1.4 (0.8; 2.2)	1.6 (0.9; 2.6)
Non-alcoholic beverages	1.4 (1.0; 2.3)	1.3 (0.9; 2.0)	2.2 (1.3; 3.8)	1.5 (1.0; 2.1)	1.5 (1.1; 2.3)	1.6 (1.1; 2.4)
Vegetables	1.3 (1.0; 1.7)	1.2 (1.0; 1.7)	1.2 (0.9; 1.6)	1.3 (1.0; 1.6)	1.3 (1.0; 1.9)	1.3 (1.0; 1.7)
Protein, EN%^c^	15 (14; 17)	15 (13; 17)	15 (13; 17)	15 (14; 17)	16 (14; 17)	15 (13; 17)
Fat, EN%^c^	45 (41; 48)	45 (41; 49)	42 (39; 46)	45 (40; 47)	45 (40; 48)	44 (41; 48)
Alcohol, gram^c^	6.0 (2.1; 16.1)	8.3 (2.9; 16.9)	6.2 (3.2; 15.4)	8.6 (2.7; 21.6)	3.4 (1.3; 11.3)	6.5 (1.8; 20.6)

Data are presented as ^a^n (%), ^b^mean ± standard deviation, or ^c^median (25th percentile; 75th percentile)

^d^ Low-GI food sources are defined as GI ≤ 55

^e^ Higher-GI food sources are defined as GI > 55

EN, energy percentage; GADA, glutamic acid decarboxylase antibody; GI, glycemic index; GL, glycemic load; MARD, moderate age-related diabetes; MOD, moderate obesity-related diabetes; OAD, oral glucose-lowering drugs; SAID, severe autoimmune diabetes; SIDD, severe insulin-deficient diabetes; SIRD, severe insulin-resistant diabetes

**Fig. 1:**
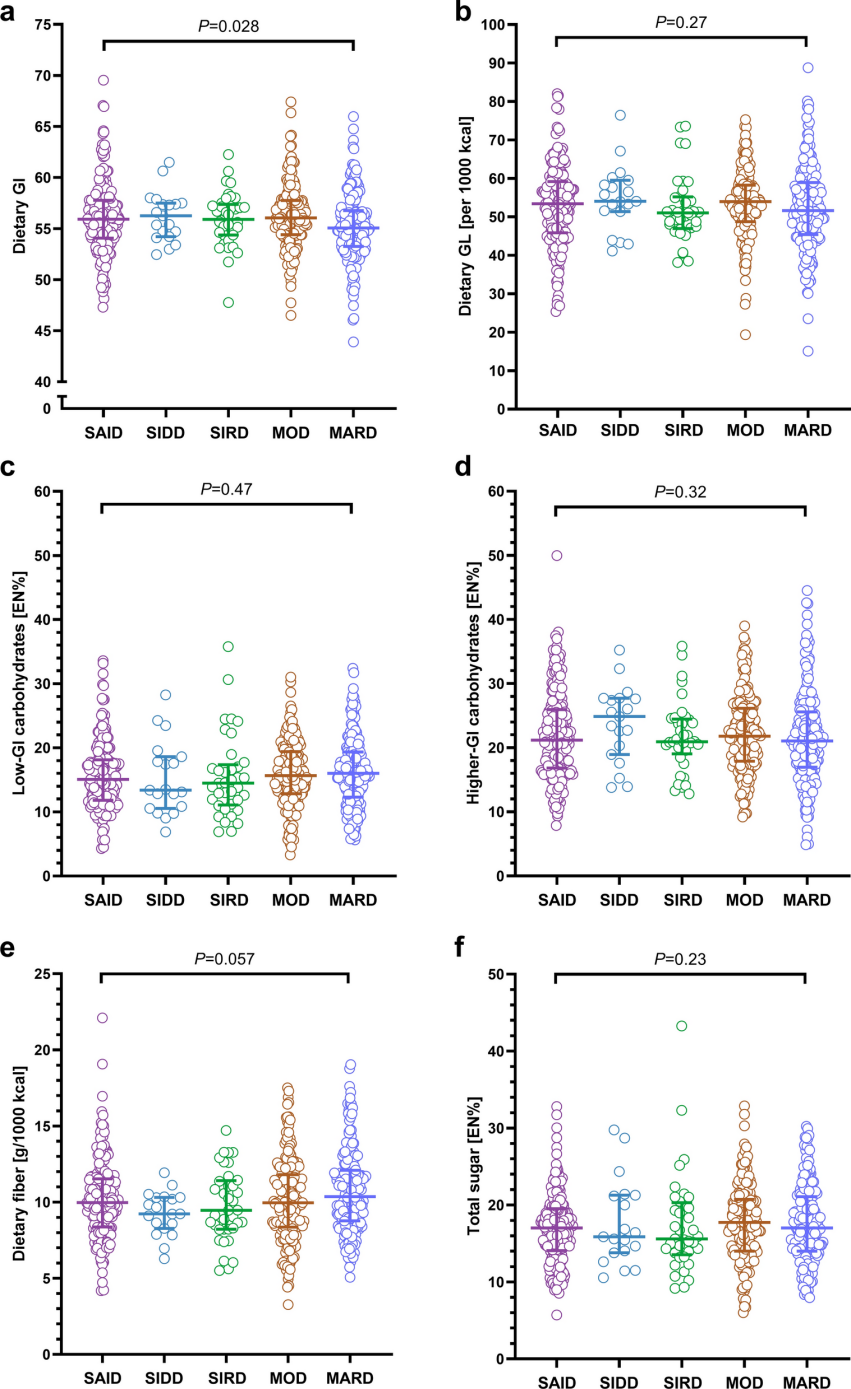
Distribution of parameters of carbohydrate quality according to subtype allocation. Scatter dot plots with median and interquartile range for **a** dietary GI, **b** dietary GL, **c** low-GI carbohydrates, **d** higher-GI carbohydrates, **e** dietary fiber, and **f** total sugar. Overall difference between subtypes assessed using Kruskal–Wallis test. EN%, energy percentage; GI, glycemic index; GL, glycemic load; MARD, moderate age-related diabetes; MOD, moderate obesity-related diabetes; SAID, severe autoimmune diabetes; SIDD, severe insulin-deficient diabetes; SIRD, severe insulin-resistant diabetes.

*Comparison of quality of carbohydrate intake between diabetes subtypes.* For dietary GI, a statistically significant, but not clinically meaningful difference was observed between the five diabetes subtypes (Fig. 1a). The remaining parameters of carbohydrate quality, ie. dietary GL, low-GI and higher-GI carbohydrates, dietary fiber, and total sugar intake, were comparable between subtypes (Fig. 1b–e). Overall, the mean dietary GL/1000 kcal was 52.9 (Table 2). Further distinguishing the quality of carbohydrate intake revealed a median intake of 16 EN% for low-GI food sources (GI < 55) and of 21 EN% for higher-GI food sources (GI ≥ 55) in the total study sample. Descriptively, people with **MOD** and **MARD** consumed the highest proportion of low-GI carbohydrates (median 16 EN%), while those with **SIDD** consumed the lowest proportion of low-GI carbohydrates (median 13 EN%) and the highest proportion of higher-GI carbohydrates (median 25 EN%) when compared between all subtypes (Table 2, Fig. 1c, d). Median dietary fiber intake was 10.0 g/1000 kcal in the total study sample and total sugar intake ranged from 16 EN% among people with **SIDD** and **SIRD** to 18 EN% among those with **MOD** (Table 2, Fig. 1f).

**Fig. 2:**
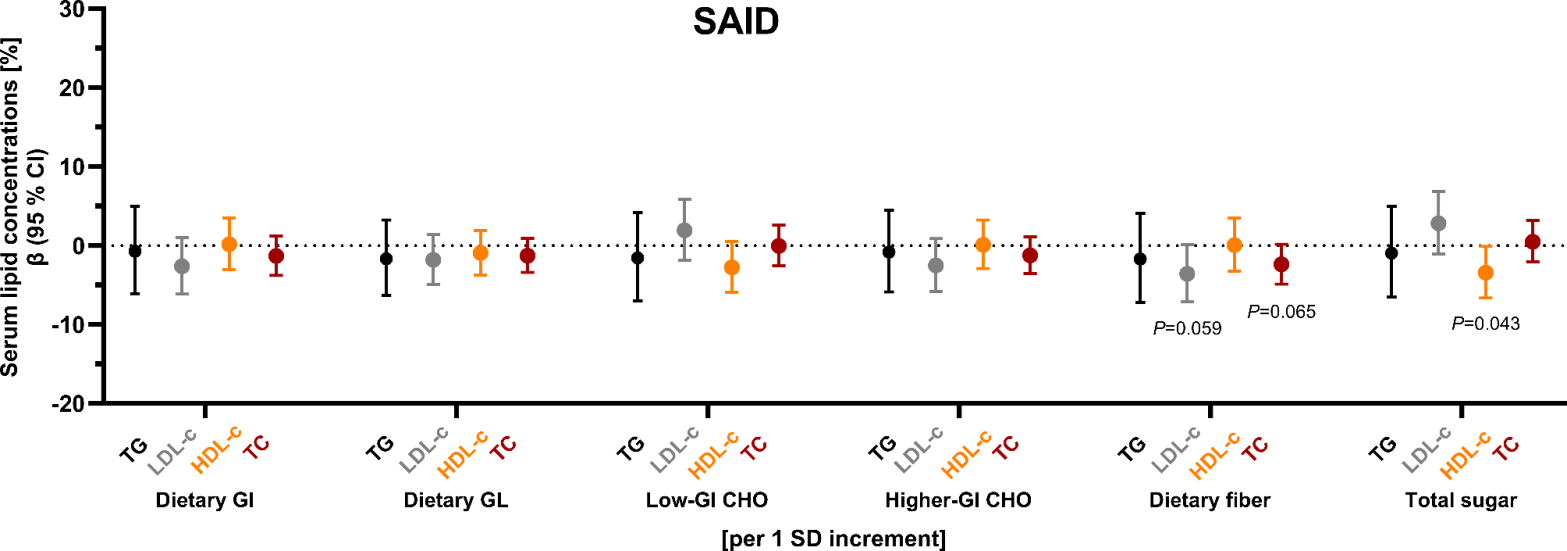
Associations of parameters of carbohydrate quality with cardiovascular risk factors among people with SAID. Regression coefficients with 95% CI for associations of carbohydrate quality with serum lipid concentrations. Adjusted for age, sex, BMI, current smoking status, total daily energy intake, socioeconomic index, partnership status, physical activity index, glucose-lowering medication, and lipid-lowering medication. Dietary GL, low-GI and higher-GI carbohydrates, dietary fiber and total sugar intake were energy adjusted using the residual method. Regression coefficients should be interpreted as follows: relative increase of the dependent variable per 1 SD increment in independent variable (Example: A 1 SD increment in dietary fiber (energy adjusted), i. e. an increase by 5.05 g, is associated with a decrease in total cholesterol by -2.5% (-4.9; -0.1) among SAID). 1 SD of dietary GI = 3.25; 1 SD of dietary GL (energy adjusted) = 23.15; 1 SD of low-GI carbohydrates (energy adjusted) = 24.87 g; 1 SD of higher-GI carbohydrates (energy adjusted) = 38.04 g; 1 SD of dietary fiber (energy adjusted) = 5.05 g; 1 SD of total sugar (energy adjusted) = 23.96 g. Black indicates triglyceride concentrations, grey indicates LDL-cholesterol concentrations, orange indicates HDL-cholesterol concentrations, and dark red indicates total cholesterol concentrations. Exact values for all comparisons are provided in Suppl. Table 1. Low-GI food sources are defined as GI≤55. Higher-GI food sources are defined as GI>55. GI, glycemic index; GL, glycemic load. CHO, carbohydrates; GI, glycemic index; GL, glycemic load; HDL-c, high-density lipoprotein cholesterol; LDL-c, low-density lipoprotein cholesterol; SAID, severe autoimmune diabetes; TC, total cholesterol; TG, triglycerides

**Fig. 3:**
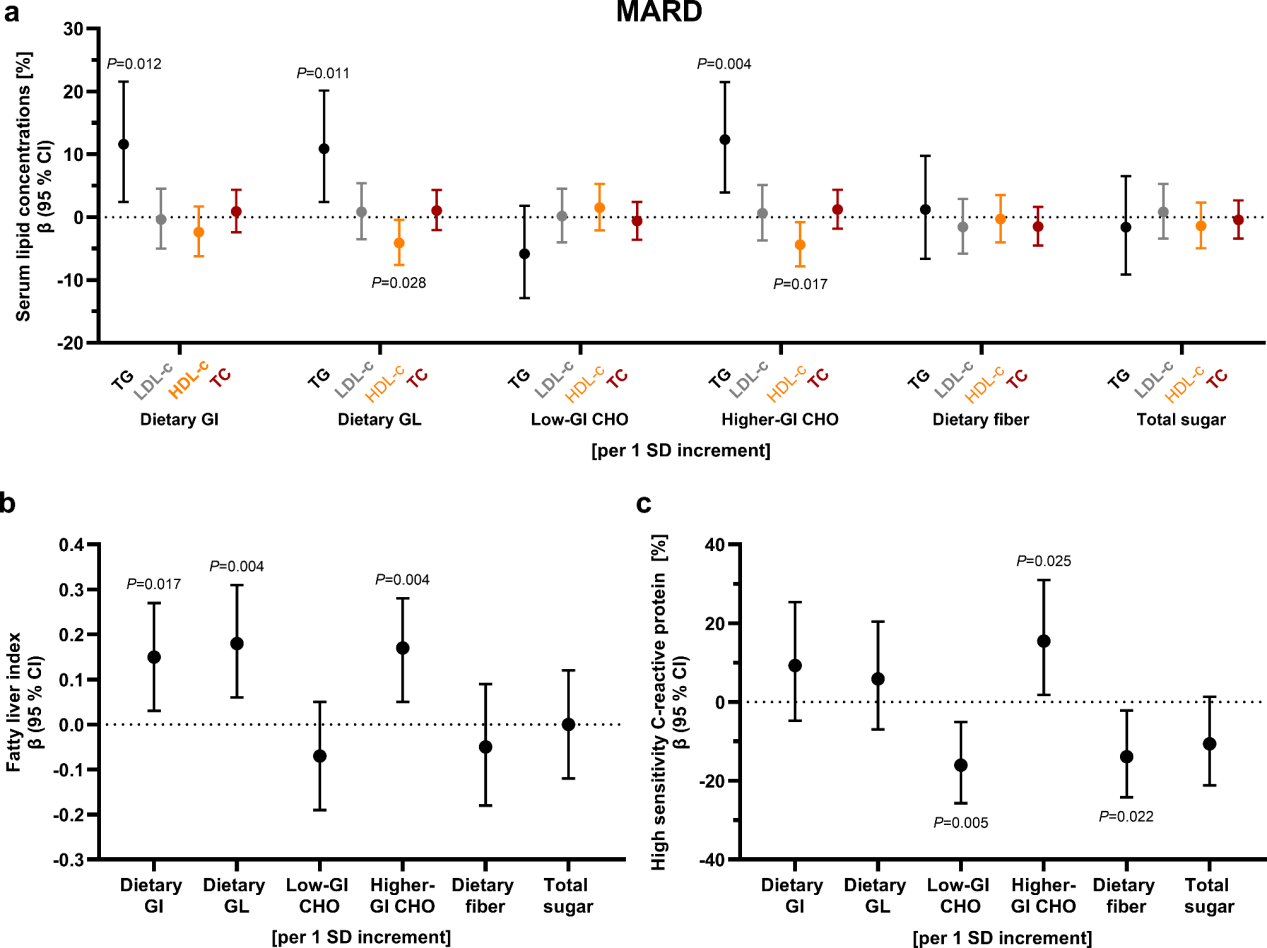
Associations of parameters of carbohydrate quality with cardiovascular risk factors among people with MARD. Regression coefficients with 95% CI for associations of carbohydrate quality with (a) serum lipid concentrations, (b) fatty liver index, and (c) hsCRP concentrations. Adjusted for age, sex, BMI, current smoking status, total daily energy intake, socioeconomic index, partnership status, physical activity index. Models including serum lipids additionally adjusted for glucose-lowering medication and lipid-lowering medication. Models including hsCRP as dependent variable additionally adjusted for glucose-lowering medication. Models including the fatty liver index additionally adjusted for total daily alcohol intake, glucose-lowering medication and lipid-lowering medication. Dietary GL, low-GI and higher-GI carbohydrates, dietary fiber and total sugar intake were energy adjusted using the residual method. Regression coefficients should be interpreted as follows: (a, c) relative increase of the dependent variable per 1 SD increment in independent variable (Example: A 1 SD increment in dietary GI, i. e. an increase by 3.25, is associated with an increase in triglycerides by 14.6% (4.1; 26.1) among MARD); (b) absolute increase of the dependent variable per 1 SD increment in independent variable (Example: A 1 SD increment in dietary GI, i. e. an increase by 3.25, is associated with an increase in fatty liver index by 0.16 (0.02; 0.30) among MARD). 1 SD of dietary GI = 3.25; 1 SD of dietary GL (energy adjusted) = 23.15; 1 SD of low-GI carbohydrates (energy adjusted) = 24.87 g; 1 SD of higher-GI carbohydrates (energy adjusted) = 38.04 g; 1 SD of dietary fiber (energy adjusted) = 5.05 g; 1 SD of total sugar (energy adjusted) = 23.96 g. Black indicates triglyceride concentrations, grey indicates LDL-cholesterol concentrations, orange indicates HDL-cholesterol concentrations, and dark red indicates total cholesterol concentrations. Exact values for all comparisons are provided in Suppl. Table 1. Low-GI food sources are defined as GI≤55. Higher-GI food sources are defined as GI>55. GI, glycemic index; GL, glycemic load. CHO, carbohydrates; GI, glycemic index; GL, glycemic load; HDL-c, high-density lipoprotein cholesterol; LDL-c, low-density lipoprotein cholesterol; MARD, moderate age-related diabetes; TC, total cholesterol; TG, triglycerides

*Associations of quality of carbohydrate intake with outcome variables and differences between diabetes subtypes.* The associations between low-GI and higher-GI carbohydrates with hsCRP and higher-GI carbohydrates with triglyceride concentrations among people with **MARD** were subtype-specific (*P*_interaction_ = 0.050, *P*_interaction_ = 0.024, and *P*_interaction_ = 0.035, respectively) (Suppl. Table 1). Thus, associations between quality of carbohydrate intake and cardiovascular risk factors are presented stratified by diabetes subtype. Among people with **SAID**, no clear associations were evident for dietary GI, GL, low- or higher-GI carbohydrates with any cardiovascular risk factor (Suppl. Table 1). However, a higher dietary fiber intake tended to be associated with lower LDL- and total cholesterol concentrations (*P* = 0.059 and *P* = 0.065, respectively), while an increase in sugar intake (energy adjusted) by 1 standard deviation (SD), ie. by 25.2 g, was associated with a decrease in HDL-cholesterol concentrations by − 3.4% (− 6.7; − 0.1) (Fig. 2). Among people with **MOD**, no clear association was evident between any of the parameters of carbohydrate quality with any cardiovascular risk factor (Suppl. Table 1). Among people with **MARD**, an increase in dietary GI and GL (energy adjusted) by 1 SD, ie. by 3.24 and 22.69, respectively, was related to an increase in triglyceride concentrations by 11.6% (2.4; 21.6) and 10.9% (2.4; 20.1), respectively, and the FLI by 0.15 (0.03; 0.27) and 0.18 (0.06; 0.31), respectively. Additionally, higher dietary GL was related to lower HDL-cholesterol concentrations (per 1 SD increment: − 4.1% (− 7.6; − 0.4)) (Fig. 3a, b, Suppl. Table 1). A higher intake of low-GI carbohydrates was associated with lower concentrations of hsCRP, whereas a higher intake of higher-GI carbohydrates was associated with higher triglyceride and hsCRP concentrations and a higher FLI, but lower HDL-cholesterol concentrations (Fig. 3, Suppl. Table 1). While a higher intake of dietary fiber was related to lower hsCRP concentrations (Fig. 3c, Suppl. Table 1), no clear associations were evident for total sugar intake with any cardiovascular risk factor among people with **MARD** (Fig. 3, Suppl. Table 1).

*Sensitivity analyses.* When including only individuals with newly-diagnosed diabetes at baseline, among people with **SAID**, the following additional associations became evident: a higher dietary GI was associated with lower LDL- and total cholesterol concentrations, a higher dietary GL was associated with lower LDL-cholesterol concentrations and a higher systolic blood pressure. The imprecisely estimated inverse associations of dietary fiber intake with LDL and total cholesterol concentrations among people with **SAID** could be replicated with higher precision in this subsample. Among people with **MARD**, the associations of higher dietary GI, dietary GL, and higher-GI carbohydrates with higher triglyceride concentrations and a higher FLI were stable in both samples. An association of higher intake of low-GI carbohydrates with lower triglyceride concentrations among people with **MARD** was confined to this subsample (Suppl. Table 2).

Estimates for the associations of carbohydrate quality parameters with cardiovascular risk factors among the three subtypes were broadly comparable when additionally considering total fat and total protein intake in the models and thus robust even after additional adjustment for these two macronutrients (Suppl. Table 3).

## Discussion

This cross-sectional analysis indicates that despite broadly comparable quality of carbohydrate intake, associations of parameters of carbohydrate quality with cardiovascular risk factors differed between the diabetes subtypes as follows. Among MARD, (i) dietary carbohydrate quality variables captured by dietary GI, GL, and intake of higher-GI carbohydrates were detrimentally related to triglyceride and HDL-cholesterol concentrations, hsCRP concentrations, and the FLI while (ii) variables indicating better carbohydrate quality (i. a. low-GI carbohydrates and dietary fiber) were associated with lower hsCRP concentrations. Similarly, among SAID (i) a higher fiber intake tended to be beneficially associated with total and LDL-cholesterol concentrations while (iii) a higher total sugar intake was associated with lower HDL-cholesterol concentrations. Interestingly, MOD, did not appear to be responsive to carbohydrate quality with respect to the investigated cardiovascular risk factors.

Our analysis is novel in that we are the first to analyze the differential relevance of parameters of carbohydrate quality for the novel diabetes subtypes. Despite the low-moderate total carbohydrate intake in our cohort with a high proportion of people with recent-onset diabetes, these participants consumed more carbohydrates from higher-GI food sources than from low-GI food sources—irrespective of the diabetes subtype allocation—so that choosing low-GI carbohydrates presents as an option to improve the carbohydrate quality of their diet. This is complemented by lower than recommended intakes of dietary fiber (median intake of 11.2 g/1000 kcal vs. recommendation of ≥ 14 g/1000 kcal for the general population) [33]. According to current dietary recommendations for people with diabetes, the focus should be on carbohydrate quality, i. a. fiber-rich and low-GI or low-GL diets, allowing for a wide range of carbohydrate intake [13, 14].

According to our observational data, people with MARD may specifically benefit from low-GI and low-GL diets for their blood lipid concentrations (ie. triglycerides and HDL-cholesterol) and for their liver fat content (related to the FLI). Also, low-GI carbohydrate choices and a high-fiber diet appear to reduce low-grade inflammation in this subtype. Although available data on the effects of low-GI or low-GL diets on concentrations of triglycerides and HDL-cholesterol among people with type 2 diabetes are inconsistent [34, 35], these diets have been shown to improve cardiometabolic risk factors such as blood lipide levels [15], which we can confirm especially for people with MARD. Our result of a direct association of GI and GL with the FLI in people with MARD is in line with the observational evidence of a direct association of GI with the odds of having non-alcoholic fatty liver disease in people with T2D [36]. In addition, lower GI/GL diets have been associated with anti-inflammatory properties in populations of or including people with type 2 diabetes, respectively [34, 37]. In contrast to people with MARD, who may especially benefit from low-GI/low-GL diets, people with SAID may rather benefit from diets high in dietary fiber and low in total sugar regarding their blood lipid profile. In people with MOD, however, no clear associations were evident for carbohydrate quality with cardiovascular risk factors. The mechanisms underlying these differences remain speculative. Due to a lack of biomarker studies, current hypotheses can only be based on e. g. differences in the variables necessary for allocation to diabetes subtypes. People with MOD, who may not be responsive to carbohydrate quality, tended to be more insulin resistant and to have a higher BMI and a higher FLI compared to those with SAID and MARD, while people with SAID, who may specifically benefit from high-fiber, low-sugar diets, were characterized by worst metabolic control [3, 4, 6]. However, the characteristics of the individuals with MOD provide no direct indication for the lack of responsiveness to dietary carbohydrate quality in this subtype. This suggests that a possible responsiveness might be masked within our study, e. g., by selective underreporting of this subgroup with a high prevalence of obesity [38]. In line with this, individuals with MOD and those with SIRD had the highest mean BMI, yet self-reported median total energy intake levels comparable to those of the other subtypes (Tables 1, 2).

To better understand possible associations between carbohydrate quality and diabetes-related complications among subtypes, further longitudinal analyses with large sample sizes and long duration of follow-up and randomized controlled interventions are needed to justify subtype-specific recommendations [5]. Together with our previous findings of differential associations between adherence to healthful dietary patterns and cardiovascular risk factors among the subtypes [5], this might point to the need for further individualization of dietary recommendations. However, evidence is insufficient to derive subtype-specific dietary recommendations with regard to carbohydrate quality and the choice of dietary patterns yet. Still, if replicated, people with MARD would specifically profit from additional advice on the selection of low-GI food choices, while the current dietary advice focusing mainly on dietary fiber and sugar may provide sufficient protection against detrimental developments in cardiovascular risk factors among people with SAID. For people with MOD, it remains unclear whether focus on carbohydrate quality may provide added benefit or if advice should focus on other modifiable risk factors.

The strengths of our study are the in-depth phenotyping of each participant as well as the precise assessment of carbohydrate intake, taking both carbohydrate quantity and quality into account. Large-scale cohort studies often derive the GI and GL from FFQs and only few studies applied questionnaires validated for these two parameters [18]. In our study, we added GI-specific questions to the established and validated FFQ, which were tailored to the food choices of the respective population for carbohydrate-rich food items of the FFQ known to be very heterogenous in their GI. In addition, this GI-extension is validated against weighted dietary records in individuals with diabetes [18]. Limitations of our study regarding the GI assessment are that the majority of currently available GI values stem from Australian and American food items with European foods still being underrepresented [11, 18] and that factors considerably influencing the GI of a food (e. g., variety, cooking methods, ripeness) are not assessed by an FFQ [18]. Limitations in terms of subtype allocation are as follows: The distinct inclusion and exclusion criteria of the GDS [16] might affect the number of people allocated to specific subtypes and thus possibly also to the low absolute number of people with SIDD and SIRD. For people with SIDD, for example, the exclusion of individuals with poor glycemic control (ie. HbA1c > 9.0%) [16] probably resulted in the omission of the most extreme SIDD cases [4–6]. Thus, due to the low absolute number of individuals with SIDD and SIRD, these two subtypes could not be considered for meaningful association analyses of carbohydrate quality with cardiovascular risk factors. However, the fact that the GDS does not uses a population-based design allows a rather large subtype with autoimmune diabetes to be analyzed. Also, especially with regard to the MOD and MARD group, the subtype distribution within the GDS is similar to cohorts with population-based recruitment [5, 39]. General limitations are that study participants of the GDS are mainly of European descent so that our results are not generalizable to other ethnic groups. Also, due to the cross-sectional study design, no causality can be inferred from our data. In addition, we used total sugar intake as added sugar intake could not be derived from the FFQ. Added sugar intake would be more informative about associations with poor diet quality and adverse metabolic outcomes [40, 41]. However, as decreasing the intake of added sugars would decrease the intake of total sugars to a similar extent [41], total sugar intake was used to complement the carbohydrate quality parameters. Also, people with recently diagnosed diabetes might have changed their diet recently and self-reported dietary intake data are prone to several biases such as recall or reporting bias. This might result in systematic misreporting of food and beverage intake due to inaccurate or incomplete recall of previous dietary intake [42]. In addition, systematic misreporting might be more pronounced in certain groups such as individuals with obesity and individuals with recently diagnosed diabetes if they report socially desirable foods, e. g. those that have been classified as healthy during dietary counselling [38, 43]. Finally, the calculation of substitution models, which were not part of our analysis plan, might provide additional important insights on the role of macronutrient replacement (ie. replacing a parameter of carbohydrate quality by a selected other macronutrient) on risk factors and disease outcomes.

## Conclusions

Taken together, despite broadly comparable quality of carbohydrate intake, differences were found between parameters of carbohydrate quality and cardiovascular risk factors across the diabetes subtypes in our cross-sectional analysis. Among MARD, higher dietary GI and GL and higher intake of higher-GI carbohydrates were detrimentally associated with the blood lipid profile, ie. higher triglyceride and lower HDL-cholesterol concentrations, with higher hsCRP concentrations, and a higher FLI, while among SAID, a higher total sugar intake was associated with lower HDL-cholesterol concentrations. People with MOD, however, showed no clear associations between carbohydrate quality and any of the investigated cardiovascular risk factors. Longitudinal analyses and randomized controlled interventions are now needed to improve the understanding of possible differential effects of carbohydrate quality on diabetes-related complications by subtypes to evaluate whether the chosen approach to reclassifying diabetes is useful in identifying groups of people who would benefit from specific nutritional approaches.

## Supplementary Information

Supplementary Material 1.

## Data Availability

Due to restrictions imposed by the ethics committee of Heinrich Heine University Düsseldorf regarding patient consent, data are available upon request. Requests for data may be sent to the last author Prof. Dr. Michael Roden (Michael.Roden@ddz.de).

## Abbreviations

BMI: Body mass index
EPIC: European Prospective Investigation into Cancer and Nutrition
FFQ: Food frequency questionnaire
FLI: Fatty liver index
GADA: Glutamic decarboxylase antibodies
GDS: German Diabetes Study
GI: Glycemic index
GL: Glycemic load
HDL: High-density lipoprotein
HOMA2-B: Homoeostasis model assessment of β-cell function
HOMA2-IR: Homoeostasis model assessment of insulin resistance
hsCRP: High sensitivity C-reactive protein
LDL: Low-density lipoprotein
MARD: Mild age-related diabetes
MOD: Mild obesity-related diabetes
SAID: Severe autoimmune diabetes
SIDD: Severe insulin-deficient diabetes
SIRD: Severe insulin-resistant diabetes
