# Supplementary material for: Associations of carbohydrate quality and cardiovascular risk factors vary among diabetes subtypes
Source: Cardiovasc Diabetol. 2025 Feb 6;24:53. doi: 10.1186/s12933-025-02580-4 (PMC11804081; doi:10.1186/s12933-025-02580-4)
Supplement: Supplementary file 1 — Supplementary Material 1. [file 12933_2025_2580_MOESM1_ESM.docx]

**SUPPLEMENTARY FIGURE**

**Supplementary Figure 1:** Flowchart

**SUPPLEMENTARY TABLES**

**Supplementary Table 1:** Associations between carbohydrate quality parameters and cardiovascular risk factors stratified by diabetes subtype

|  | **Total**  (n=638) | **SAID**  (n=245) | **MOD**  (n=193) | **MARD**  (n=200) | *P*_int_ |
| --- | --- | --- | --- | --- | --- |
|  | ß (95% CI) | ß (95% CI) | ß (95% CI) | ß (95% CI) |  |
| **Dietary GI** |  |  |  |  |  |
| Triglycerides [mg/dL]^b^ | 3.9 (-0.4; 8.4) | -0.7 (-6.1; 5.0) | 0.0 (-8.2; 9.0) | **11.6 (2.4; 21.6)*** | 0.11 |
| LDL-cholesterol [mg/dL]^b^ | -0.8 (-3.2; 1.6) | -2.6 (-6.2; 1.0) | 0.9 (-3.7; 5.8) | -0.3 (-5.0; 4.5) | 0.34 |
| HDL-cholesterol [mg/dL]^b^ | -0.6 (-2.7; 1.5) | 0.2 (-3.1; 3.5) | 0.5 (-3.2; 4.4) | -2.4 (-6.2; 1.7) | 0.47 |
| Total cholesterol [mg/dL]^b^ | 0.1 (-1.6; 1.8) | -1.3 (-3.8; 1.2) | 0.7 (-2.7; 4.2) | 0.9 (-2.4; 4.4) | 0.28 |
| hsCRP [mg/dL]^b^ | 1.6 (-5.5; 9.2) | -4.2 (-15.6; 8.7) | 0.2 (-11.4; 13.4) | 9.3 (-4.8; 25.3) | 0.21 |
| Diastolic blood pressure [mmHg]^a^ | 0.0 (-0.8; 0.8) | 0.5 (-0.7; 1.6) | 0.1 (-1.5; 1.8) | -0.6 (-2.1; 1.0) | 0.92 |
| Systolic blood pressure [mmHg]^a^ | 0.0 (-1.2; 1.3) | 0.7 (-10.0; 2.4) | 0.9 (-1.4; 3.2) | -1.2 (-3.9; 1.6) | 0.25 |
| Fatty liver index^a^ | **0.09 (0.02; 0.16)*** | 0.05 (-0.04; 0.15) | 0.03 (-0.12; 0.17) | **0.15 (0.03; 0.27)*** | 0.52 |
| **Dietary GL** (energy adjusted) |  |  |  |  |  |
| Triglycerides [mg/dL]^b^ | 2.6 (-1.4; 6.8) | -1.7 (-6.3; 3.2) | 1.2 (-8.7; 12.1) | **10.9 (2.4; 20.1)*** | 0.055 |
| LDL-cholesterol [mg/dL]^b^ | -0.5 (-2.8; 1.8) | -1.8 (-4.9; 1.4) | 2.1 (-3.5; 8.0) | 0.8 (-3.5; 5.4) | 0.20 |
| HDL-cholesterol [mg/dL]^b^ | **-2.1 (-4.0; -0.2)*** | -0.9 (-3.7; 1.9) | -2.9 (-7.1; 1.5) | **-4.1 (-7.6; -0.4)*** | 0.26 |
| Total cholesterol [mg/dL]^b^ | -0.1 (-1.7; 1.5) | -1.3 (-3.4; 0.9) | 1.6 (-2.4; 5.9) | 1.1 (-2.1; 4.3) | 0.20 |
| hsCRP [mg/dL]^b^ | -2.0 (-8.6; 5.0) | -6.8 (-16.5; 4.1) | -7.3 (-19.9; 7.4) | 5.9 (-6.9; 20.4) | 0.21 |
| Diastolic blood pressure [mmHg]^a^ | -0.1 (-0.9; 0.6) | 0.2 (-0.8; 1.3) | 0.2 (-1.7; 2.2) | -0.8 (-2.3; 0.6) | 0.48 |
| Systolic blood pressure [mmHg]^a^ | 0.1 (-1.1; 1.3) | 0.9 (-0.5; 2.4) | -0.1 (-2.8; 2.6) | -0.5 (-3.1; 2.1) | 0.39 |
| Fatty liver index^a^ | 0.06 (-0.01; 0.13) | 0.00 (-0.09; 0.08) | 0.05 (-0.13; 0.23) | **0.18 (0.06; 0.31)**** | 0.084 |
| **Low-GI carbohydrates^c^** (energy adjusted) |  |  |  |  |  |
| Triglycerides [mg/dL]^b^ | -3.6 (-7.4; 0.4) | -1.6 (-7.0; 4.2) | -0.7 (-8.1; 7.4) | -5.8 (-12.9; 1.8) | 0.46 |
| LDL-cholesterol [mg/dL]^b^ | -0.3 (-2.6; 2.0) | 1.9 (-1.8; 5.8) | -1.6 (-5.7; 2.7) | 0.2 (-4.0; 4.5) | 0.62 |
| HDL-cholesterol [mg/dL]^b^ | -0.2 (-2.2; 1.8) | -2.8 (-5.9; 0.5) | -0.3 (-3.6; 3.2) | 1.5 (-2.1; 5.3) | 0.20 |
| Total cholesterol [mg/dL]^b^ | -0.6 (-2.2; 1.0) | 0.0 (-2.6; 2.6) | -0.3 (-3.3; 2.9) | -0.6 (-3.6; 2.4) | 0.92 |
| hsCRP [mg/dL]^b^ | -6.4 (-12.7; 0.4) | 1.7 (-10.6; 15.7) | -3.2 (-13.4; 8.3) | **-16.0 (-25.7; -5.1)**** | **0.050** |
| Diastolic blood pressure [mmHg]^a^ | -0.2 (-1.0; 0.6) | 0.3 (-0.9; 1.5) | -0.2 (-1.7; 1.3) | -0.8 (-2.2; 0.6) | 0.32 |
| Systolic blood pressure [mmHg]^a^ | -0.4 (-1.6; 0.8) | 0.7 (-1.1; 2.4) | -1.5 (-3.6; 0.5) | -0.6 (-3.1; 1.9) | 0.36 |
| Fatty liver index^a^ | -0.05 (-0.12; 0.01) | -0.02 (-0.11; 0.07) | 0.01 (-0.12; 0.14) | -0.07 (-0.19; 0.05) | 0.84 |
| **Higher-GI carbohydrates^d^** (energy adjusted) |  |  |  |  |  |
| Triglycerides [mg/dL]^b^ | 4.1 (-0.1; 8.4) | -0.8 (-5.9; 4.5) | 1.3 (-7.9; 11.3) | **12.4 (3.9; 21.5)**** | **0.035** |
| LDL-cholesterol [mg/dL]^b^ | 0.0 (-2.3; 2.3) | -2.5 (-5.8; 0.9) | 3.2 (-2.0; 8.7) | 0.6 (-3.7; 5.1) | 0.10 |
| HDL-cholesterol [mg/dL]^b^ | -1.9 (-3.8; 0.1) | 0.1 (-2.9; 3.2) | -2.0 (-5.9; 2.1) | **-4.4 (-7.8; -0.8)*** | 0.12 |
| Total cholesterol [mg/dL]^b^ | 0.3 (-1.4; 1.9) | -1.2 (-3.5; 1.1) | 1.6 (-2.2; 5.5) | 1.2 (-1.9; 4.4) | 0.18 |
| hsCRP [mg/dL]^b^ | 1.5 (-5.3; 8.9) | -7.3 (-17.6; 4.2) | -3.5 (-15.7; 10.5) | **15.4 (1.8; 30.9)*** | **0.024** |
| Diastolic blood pressure [mmHg]^a^ | 0.0 (-0.8; 0.8) | 0.0 (-1.0; 1.1) | 0.3 (-1.5; 2.0) | -0.1 (-1.5; 1.3) | 0.95 |
| Systolic blood pressure [mmHg]^a^ | 0.3 (-0.9; 1.5) | 0.5 (-1.1; 2.1) | 1.0 (-1.4; 3.5) | 0.2 (-2.3; 2.8) | 0.98 |
| Fatty liver index^a^ | 0.07 (0.00; 0.13) | -0.01 (-0.09; 0.08) | 0.02 (-0.14; 0.18) | **0.17 (0.05; 0.28)**** | 0.088 |
| **Dietary fiber** (energy adjusted) |  |  |  |  |  |
| Triglycerides [mg/dL]^b^ | -2.1 (-6.1; 2.1) | -1.7 (-7.2; 4.1) | -3.6 (-11.2; 4.7) | 1.2 (-6.7; 9.8) | 0.76 |
| LDL-cholesterol [mg/dL]^b^ | -1.6 (-4.0; 0.7) | -3.6 (-7.1; 0.1) | 0.7 (-3.7; 5.4) | -1.5 (-5.8; 2.9) | 0.15 |
| HDL-cholesterol [mg/dL]^b^ | 0.0 (-2.0; 2.1) | 0.1 (-3.3; 3.5) | 0.7 (-2.9; 4.3) | -0.3 (-4.0; 3.5) | 0.98 |
| Total cholesterol [mg/dL]^b^ | -1.5 (-3.1; 0.2) | -2.4 (-4.9; 0.2) | 0.4 (-2.8; 3.8) | -1.5 (-4.5; 1.6) | 0.32 |
| hsCRP [mg/dL]^b^ | -5.0 (-11.5; 2.1) | -5.6 (-17.1; 7.4) | 6.4 (-5.4; 19.7) | **-13.9 (-24.2; -2.2)*** | 0.053 |
| Diastolic blood pressure [mmHg]^a^ | -0.2 (-1.0; 0.6) | -0.6 (-1.8; 0.6) | 0.2 (-1.3; 1.8) | -0.5 (-2.0; 1.0) | 0.76 |
| Systolic blood pressure [mmHg]^a^ | -0.2 (-1.5; 1.0) | -0.3 (-2.0; 1.4) | 0.0 (-2.2; 2.2) | -0.4 (-3.0; 2.2) | 0.81 |
| Fatty liver index^a^ | -0.04 (-0.11; 0.03) | -0.01 (-0.11; 0.08) | -0.01 (-0.15; 0.13) | -0.05 (-0.18; 0.09) | 0.92 |
| **Total sugar** (energy adjusted) |  |  |  |  |  |
| Triglycerides [mg/dL]^b^ | -0.4 (-4.3; 3.7) | -1.0 (-6.5; 5.0) | 2.1 (-5.0; 9.8) | -1.6 (-9.1; 6.5) | 0.80 |
| LDL-cholesterol [mg/dL]^b^ | 0.3 (-2.0; 2.7) | 2.8 (-1.1; 6.8) | -1.4 (-5.3; 2.5) | 0.8 (-3.4; 5.3) | 0.40 |
| HDL-cholesterol [mg/dL]^b^ | -1.8 (-3.8; 0.1) | **-3.4 (-6.7; -0.1)*** | -1.5 (-4.6; 1.6) | -1.4 (-4.9; 2.3) | 0.58 |
| Total cholesterol [mg/dL]^b^ | -0.1 (-1.7; 1.6) | 0.5 (-2.1; 3.2) | 0.1 (-2.8; 3.0) | -0.4 (-3.4; 2.7) | 0.90 |
| hsCRP [mg/dL]^b^ | -2.7 (-9.3; 4.2) | 1.7 (-10.9; 16) | -0.1 (-9.9; 10.8) | -10.6 (-21.2; 1.3) | 0.26 |
| Diastolic blood pressure [mmHg]^a^ | -0.1 (-0.9; 0.6) | 0.3 (-0.9; 1.5) | 0.1 (-1.2; 1.5) | -0.9 (-2.3; 0.6) | 0.25 |
| Systolic blood pressure [mmHg]^a^ | -0.1 (-1.3; 1.1) | 0.9 (-0.9; 2.6) | -1.3 (-3.3; 0.6) | 0.0 (-2.5; 2.5) | 0.26 |
| Fatty liver index^a^ | -0.01 (-0.08; 0.05) | -0.01 (-0.11; 0.08) | 0.01 (-0.1; 0.13) | 0.00 (-0.12; 0.12) | 0.94 |

Due to the low absolute number of participants with SIDD and SIRD, these two subtypes were excluded from association and interaction analyses.

Adjusted for age, sex, BMI, current smoking status, total daily energy intake, socioeconomic index, partnership status, physical activity index. Models including serum lipids additionally adjusted for glucose-lowering medication and lipid-lowering medication. Models including hsCRP as dependent variable additionally adjusted for glucose-lowering medication. Models including blood pressure as dependent variable additionally adjusted for antihypertensive medication. Models including fatty liver index as dependent variable additionally adjusted for total daily alcohol intake, glucose-lowering medication and lipid-lowering medication. Dietary GL, Low-GI and higher-GI carbohydrates, dietary fiber and total sugar intake were energy adjusted using the residual method.

Regression coefficients should be interpreted as follows: ^a^ absolute increase of the dependent variable per 1 SD increment in the independent variable (Example: A 1 SD increment in dietary GI, i. e. an increase by 3.24, is associated with a decrease in systolic blood pressure by -1.2 mmHg (-3.9; 1.6) among MARD); ^b^ relative increase of the dependent variable per 1 SD increment in independent variable (Example: A 1 SD increment in dietary GI, i. e. an increase by 3.24, is associated with an increase in triglycerides by 11.6% (2.4; 21.6) among MARD). 1 SD of dietary GI = 3.24; 1 SD of dietary GL (energy adjusted) = 22.69; 1 SD of low-GI carbohydrates (energy adjusted) = 27.04 g; 1 SD of higher-GI carbohydrates (energy adjusted) = 38.67 g; 1 SD of dietary fiber (energy adjusted) = 6.36 g; 1 SD of total sugar (energy adjusted) = 25.20 g.

^c^ Low-GI food sources are defined as GI≤55. ^d^ Higher-GI food sources are defined as GI>55.

**P* <0.05, **Bonferroni corrected *P* <0.0083 (=0.05/m with m=6 for number of parameters of carbohydrate quality).

CI, confidence interval; HDL, high-density lipoprotein; hsCRP, high-sensitivity C-reactive protein; LDL, low-density lipoprotein; MARD, moderate age-related diabetes; MOD, moderate obesity-related diabetes; OAD, oral glucose-lowering drugs; *P*­_int_, *P*-value for interaction with parameters of carbohydrate quality *diabetes subtypes as interaction term; SAID, severe autoimmune diabetes.

**Supplementary Table 2:** Associations between carbohydrate quality parameters and cardiovascular risk factors stratified by diabetes subtype only including individuals at baseline examination

|  | **Total**  (n=447) | **SAID**  (n=175) | **MOD**  (n=133) | **MARD**  (n=139) |
| --- | --- | --- | --- | --- |
|  | ß (95% CI) | ß (95% CI) | ß (95% CI) | ß (95% CI) |
| **Dietary GI** |  |  |  |  |
| Triglycerides [mg/dL]^b^ | 3.1 (-1.9; 8.3) | -2.8 (-8.8; 3.6) | 1.6 (-9.8; 14.5) | **14.6 (4.1; 26.1)**** |
| LDL-cholesterol [mg/dL]^b^ | -2.4 (-5.2; 0.5) | **-4.8 (-8.8; -0.6)*** | -1.5 (-8.2; 5.7) | 0.2 (-5.2; 5.8) |
| HDL-cholesterol [mg/dL]^b^ | -1.6 (-3.9; 0.8) | -1.6 (-5.2; 2.2) | -2.4 (-7.3; 2.8) | -1.6 (-5.7; 2.8) |
| Total cholesterol [mg/dL]^b^ | -1.2 (-3.2; 0.8) | **-3.2 (-6.0; -0.4)*** | -0.9 (-6.0; 4.5) | 1.4 (-2.4; 5.3) |
| hsCRP [mg/dL]^b^ | 1.5 (-6.9; 10.8) | -7.8 (-20.7; 7.1) | 10.8 (-6.7; 31.7) | 10.7 (-4.5; 28.4) |
| Diastolic blood pressure [mmHg]^a^ | 0.2 (-0.8; 1.2) | 0.8 (-0.6; 2.2) | 1.1 (-1.3; 3.5) | -0.7 (-2.5; 1.1) |
| Systolic blood pressure [mmHg]^a^ | 0.3 (-1.3; 1.9) | 1.3 (-0.7; 3.3) | 2.6 (-0.8; 5.9) | -1.8 (-5.1; 1.4) |
| Fatty liver index^a^ | 0.05 (-0.03; 0.13) | 0.02 (-0.10; 0.13) | 0.00 (-0.22; 0.21) | **0.16 (0.01; 0.30)*** |
| **Dietary GL** (energy adjusted) |  |  |  |  |
| Triglycerides [mg/dL]^b^ | 1.9 (-2.7; 6.7) | -2.5 (-7.8; 3.1) | 0.9 (-11.4; 14.9) | **11.5 (1.6; 22.4)*** |
| LDL-cholesterol [mg/dL]^b^ | -1.5 (-4.2; 1.2) | **-3.7 (-7.3; -0.1)*** | 1.8 (-5.7; 9.9) | 1.9 (-3.3; 7.4) |
| HDL-cholesterol [mg/dL]^b^ | -2.2 (-4.4; 0.0) | -1.2 (-4.4; 2.1) | -4.9 (-10; 0.6) | -3.4 (-7.3; 0.6) |
| Total cholesterol [mg/dL]^b^ | -0.7 (-2.6; 1.3) | -2.3 (-4.7; 0.2) | 1.8 (-3.9; 7.8) | 1.6 (-2.0; 5.4) |
| hsCRP [mg/dL]^b^ | -0.4 (-8.2; 8.1) | -4.4 (-16.2; 9) | -7.4 (-23.3; 11.7) | 9.5 (-5.2; 26.4) |
| Diastolic blood pressure [mmHg]^a^ | 0.2 (-0.7; 1.1) | 0.8 (-0.4; 2.1) | 0.7 (-1.9; 3.4) | -0.9 (-2.6; 0.8) |
| Systolic blood pressure [mmHg]^a^ | 0.8 (-0.7; 2.2) | **2.0 (0.3; 3.8)*** | 0.4 (-3.4; 4.1) | -0.5 (-3.6; 2.6) |
| Fatty liver index^a^ | 0.03 (-0.04; 0.11) | -0.03 (-0.13; 0.07) | 0.06 (-0.18; 0.29) | **0.18 (0.04; 0.32)*** |
| **Low-GI carbohydrates^c^** (energy adjusted) |  |  |  |  |
| Triglycerides [mg/dL]^b^ | -3.6 (-8.1; 1.0) | -1.6 (-8.2; 5.5) | -0.3 (-9.0; 9.3) | **-9.1 (-17; -0.5)*** |
| LDL-cholesterol [mg/dL]^b^ | 0.5 (-2.3; 3.4) | 0.6 (-4.0; 5.5) | 1.5 (-3.9; 7.1) | 1.3 (-3.7; 6.6) |
| HDL-cholesterol [mg/dL]^b^ | 0.2 (-2.1; 2.6) | -0.5 (-4.5; 3.7) | 1.4 (-2.5; 5.6) | 0.0 (-4.0; 4.1) |
| Total cholesterol [mg/dL]^b^ | 0.3 (-1.6; 2.3) | -0.1 (-3.3; 3.1) | 2.1 (-2.0; 6.3) | -0.3 (-3.8; 3.3) |
| hsCRP [mg/dL]^b^ | -4.2 (-11.9; 4.1) | 2.6 (-12.9; 20.8) | -10.7 (-21.6; 1.9) | -6.7 (-18.9; 7.5) |
| Diastolic blood pressure [mmHg]^a^ | -0.3 (-1.3; 0.6) | 0.5 (-1.0; 2.1) | -0.8 (-2.6; 1.1) | -1.0 (-2.8; 0.7) |
| Systolic blood pressure [mmHg]^a^ | -0.4 (-1.9; 1.1) | 0.5 (-1.7; 2.7) | -2.0 (-4.5; 0.6) | -0.1 (-3.2; 3.0) |
| Fatty liver index^a^ | -0.04 (-0.12; 0.04) | -0.08 (-0.20; 0.04) | 0.04 (-0.12; 0.20) | -0.06 (-0.20; 0.07) |
| **Higher-GI carbohydrates^d^** (energy adjusted) |  |  |  |  |
| Triglycerides [mg/dL]^b^ | 3.8 (-1.0; 8.8) | -1.6 (-7.4; 4.6) | 1.2 (-10.3; 14.1) | **15.6 (5.6; 26.7)**** |
| LDL-cholesterol [mg/dL]^b^ | -1.2 (-4.0; 1.6) | -3.7 (-7.6; 0.4) | 1.2 (-5.7; 8.7) | 1.0 (-4.1; 6.4) |
| HDL-cholesterol [mg/dL]^b^ | -2.3 (-4.5; 0.0) | -1.4 (-4.8; 2.2) | -4.9 (-9.7; 0.1) | -3.2 (-7.1; 0.8) |
| Total cholesterol [mg/dL]^b^ | -0.6 (-2.5; 1.4) | -2.1 (-4.8; 0.6) | 0.3 (-4.9; 5.8) | 1.6 (-2.1; 5.3) |
| hsCRP [mg/dL]^b^ | 1.6 (-6.5; 10.4) | -4.8 (-17.5; 9.8) | 2.1 (-14.2; 21.6) | 11.4 (-3.4; 28.6) |
| Diastolic blood pressure [mmHg]^a^ | 0.3 (-0.6; 1.3) | 0.6 (-0.8; 1.9) | 1.0 (-1.4; 3.4) | -0.1 (-1.9; 1.6) |
| Systolic blood pressure [mmHg]^a^ | 0.9 (-0.5; 2.4) | 1.8 (-0.1; 3.8) | 1.6 (-1.7; 5.0) | 0.0 (-3.1; 3.2) |
| Fatty liver index^a^ | 0.05 (-0.03; 0.13) | 0.00 (-0.11; 0.1) | 0.02 (-0.20; 0.23) | **0.18 (0.04; 0.31)*** |
| **Dietary fiber** (energy adjusted) |  |  |  |  |
| Triglycerides [mg/dL]^b^ | -4.3 (-8.7; 0.3) | -3.5 (-9.8; 3.3) | -5.8 (-14.0; 3.2) | -1.1 (-10.1; 8.9) |
| LDL-cholesterol [mg/dL]^b^ | -2.3 (-5.0; 0.5) | **-5.8 (-10.0; -1.5)*** | -0.5 (-5.7; 5.1) | 0.9 (-4.3; 6.4) |
| HDL-cholesterol [mg/dL]^b^ | 0.4 (-1.9; 2.7) | 0.3 (-3.7; 4.3) | 1.6 (-2.4; 5.8) | -0.7 (-4.8; 3.6) |
| Total cholesterol [mg/dL]^b^ | -2.0 (-3.9; 0.0) | **-3.4 (-6.3; -0.4)*** | -0.6 (-4.6; 3.6) | -0.7 (-4.3; 3.1) |
| hsCRP [mg/dL]^b^ | -2.9 (-10.7; 5.5) | -6.0 (-19.8; 10.2) | 5.8 (-7.3; 20.9) | -3.3 (-16.6; 12) |
| Diastolic blood pressure [mmHg]^a^ | 0.0 (-1.0; 0.9) | -0.7 (-2.2; 0.8) | 1.0 (-0.9; 2.9) | -0.4 (-2.2; 1.4) |
| Systolic blood pressure [mmHg]^a^ | 0.6 (-0.9; 2.1) | 0.4 (-1.8; 2.5) | 0.9 (-1.8; 3.6) | 0.3 (-2.8; 3.5) |
| Fatty liver index^a^ | -0.07 (-0.15; 0.01) | -0.04 (-0.16; 0.09) | -0.04 (-0.21; 0.13) | -0.07 (-0.23; 0.09) |
| **Total sugar** (energy adjusted) |  |  |  |  |
| Triglycerides [mg/dL]^b^ | -0.2 (-4.7; 4.5) | 0.1 (-6.6; 7.3) | 4.3 (-4.6; 14.0) | -4.4 (-12.4; 4.3) |
| LDL-cholesterol [mg/dL]^b^ | 1.3 (-1.4; 4.2) | 2.2 (-2.5; 7.1) | 2.0 (-3.3; 7.5) | 1.1 (-3.7; 6.1) |
| HDL-cholesterol [mg/dL]^b^ | -1.6 (-3.8; 0.6) | -1.9 (-5.8; 2.2) | -1.4 (-5.2; 2.5) | -1.8 (-5.5; 2.1) |
| Total cholesterol [mg/dL]^b^ | 0.9 (-1.0; 2.9) | 0.7 (-2.5; 3.9) | 3.0 (-1.0; 7.1) | -0.4 (-3.7; 3.0) |
| hsCRP [mg/dL]^b^ | 0.0 (-7.8; 8.5) | 3.2 (-12.4; 21.5) | -6.6 (-17.9; 6.2) | -0.8 (-13.3; 13.6) |
| Diastolic blood pressure [mmHg]^a^ | -0.1 (-1.0; 0.8) | 1.0 (-0.6; 2.5) | -0.3 (-2.1; 1.5) | -0.9 (-2.6; 0.7) |
| Systolic blood pressure [mmHg]^a^ | -0.3 (-1.7; 1.2) | 1.1 (-1.1; 3.3) | -1.7 (-4.2; 0.8) | -0.2 (-3.2; 2.7) |
| Fatty liver index^a^ | 0.01 (-0.06; 0.09) | -0.05 (-0.17; 0.08) | 0.08 (-0.08; 0.24) | 0.01 (-0.12; 0.14) |

Due to the low absolute number of participants with SIDD and SIRD, these two subtypes were excluded from association and interaction analyses.

Adjusted for age, sex, BMI, current smoking status, total daily energy intake, socioeconomic index, partnership status, physical activity index. Models including serum lipids additionally adjusted for glucose-lowering medication and lipid-lowering medication. Models including hsCRP as dependent variable additionally adjusted for glucose-lowering medication. Models including blood pressure as dependent variable additionally adjusted for antihypertensive medication. Models including fatty liver index as dependent variable additionally adjusted for total daily alcohol intake, glucose-lowering medication and lipid-lowering medication. Dietary GL, low-GI and higher-GI carbohydrates, dietary fiber and total sugar intake were energy adjusted using the residual method.

Regression coefficients should be interpreted as follows: ^a^ absolute increase of the dependent variable per 1 SD increment in independent variable (Example: A 1 SD increment in dietary GI, i. e. an increase by 3.25, is associated with a decrease in systolic blood pressure by -1.8 mmHg (-5.1; 1.4) among MARD); ^b^ relative increase of the dependent variable per 1 SD increment in independent variable (Example: A 1 SD increment in dietary GI, i. e. an increase by 3.24, is associated with an increase in triglycerides by 14.6% (4.1; 26.1) among MARD). 1 SD of dietary GI = 3.25; 1 SD of dietary GL (energy adjusted) = 23.15; 1 SD of low-GI carbohydrates (energy adjusted) = 24.87 g; 1 SD of higher GI carbohydrates (energy adjusted) = 38.04 g; 1 SD of dietary fiber (energy adjusted) = 6.11 g; 1 SD of total sugar (energy adjusted) = 23.96 g.

^c^ Low-GI food sources are defined as GI≤55. ^d^ Higher-GI food sources are defined as GI>55.

**P* <0.05. **Bonferroni corrected *P* <0.0083 (=0.05/m with m=6 for number of parameters of carbohydrate quality).

CI, confidence interval; HDL, high-density lipoprotein; hsCRP, high-sensitivity C-reactive protein; LDL, low-density lipoprotein; MARD, moderate age-related diabetes; MOD, moderate obesity-related diabetes; OAD, oral glucose-lowering drugs; SAID, severe autoimmune diabetes.

**Supplementary Table 3:** Associations between carbohydrate quality parameters and cardiovascular risk factors stratified by diabetes subtype and additionally considering protein and fat intake as confounders

|  | **Total**  (n=638) | **SAID**  (n=245) | **MOD**  (n=193) | **MARD**  (n=200) |
| --- | --- | --- | --- | --- |
|  |  | ß (95% CI) | ß (95% CI) | ß (95% CI) |
| **Dietary GI** |  |  |  |  |
| Triglycerides [mg/dL]^b^ | 3.3 (-1.1; 7.9) | -0.7 (-6.1; 5.2) | -1.0 (-9.4; 8.2) | 8.9 (-0.8; 19.6) |
| LDL-cholesterol [mg/dL]^b^ | -0.8 (-3.3; 1.7) | -2.5 (-6.1; 1.3) | 0.0 (-4.7; 5.0) | 0.3 (-4.7; 5.7) |
| HDL-cholesterol [mg/dL]^b^ | -0.6 (-2.8; 1.5) | 0.0 (-3.2; 3.5) | 0.5 (-3.4; 4.4) | -1.7 (-6.0; 2.7) |
| Total cholesterol [mg/dL]^b^ | -0.1 (-1.8; 1.7) | -1.3 (-3.8; 1.3) | -0.1 (-3.6; 3.5) | 1.1 (-2.5; 4.9) |
| hsCRP [mg/dL]^b^ | 1.7 (-5.7; 9.7) | -3.4 (-15.1; 9.9) | 0.8 (-11.2; 14.4) | 5.6 (-9.2; 22.7) |
| Diastolic blood pressure [mmHg]^a^ | 0.0 (-0.8; 0.9) | 0.5 (-0.7; 1.7) | 0.0 (-1.7; 1.7) | -0.4 (-2.1; 1.3) |
| Systolic blood pressure [mmHg]^a^ | -0.2 (-1.5; 1.1) | 0.6 (-1.2; 2.3) | 0.8 (-1.6; 3.1) | -1.9 (-5.0; 1.1) |
| Fatty liver index^a^ | **0.08 (0.01; 0.15)*** | 0.06 (-0.04; 0.16) | 0.01 (-0.14; 0.16) | 0.10 (-0.04; 0.24) |
| **Dietary GL** (energy adjusted) |  |  |  |  |
| Triglycerides [mg/dL]^b^ | 2.6 (-3.1; 8.6) | -2.7 (-9.8; 4.9) | -3.5 (-16; 10.9) | 8.9 (-1.6; 20.6) |
| LDL-cholesterol [mg/dL]^b^ | 0.1 (-3.1; 3.5) | -2.1 (-6.8; 2.9) | 1.6 (-5.9; 9.6) | 2.4 (-3.2; 8.4) |
| HDL-cholesterol [mg/dL]^b^ | **-4.0 (-6.6; -1.2)**** | -2.8 (-7.0; 1.6) | -4.2 (-9.9; 1.7) | **-4.8 (-9.3; -0.2)*** |
| Total cholesterol [mg/dL]^b^ | -0.3 (-2.6; 2.0) | -1.9 (-5.1; 1.5) | -0.8 (-6.2; 4.8) | 1.6 (-2.4; 5.7) |
| hsCRP [mg/dL]^b^ | -2.1 (-11.3; 8.0) | -6.0 (-20.8; 11.5) | -3.2 (-20.6; 18) | -1.1 (-16.2; 16.6) |
| Diastolic blood pressure [mmHg]^a^ | 0.0 (-1.1; 1.1) | 0.3 (-1.3; 1.9) | 0.1 (-2.6; 2.7) | -0.7 (-2.6; 1.2) |
| Systolic blood pressure [mmHg]^a^ | -0.5 (-2.2; 1.2) | 0.9 (-1.4; 3.2) | -0.6 (-4.2; 3.1) | -1.7 (-5.0; 1.6) |
| Fatty liver index^a^ | **0.17 (0.00; 0.34)*** | 0.06 (-0.17; 0.29) | 0.01 (-0.33; 0.35) | 0.36 (-0.01; 0.72) |
| **Low-GI carbohydrates^c^** (energy adjusted) |  |  |  |  |
| Triglycerides [mg/dL]^b^ | -3.6 (-7.5; 0.4) | -1.4 (-7.0; 4.6) | -1.2 (-9.1; 7.5) | -5.3 (-12.4; 2.3) |
| LDL-cholesterol [mg/dL]^b^ | 0.0 (-2.4; 2.3) | 2.4 (-1.5; 6.5) | -1.1 (-5.6; 3.5) | 0.0 (-4.2; 4.4) |
| HDL-cholesterol [mg/dL]^b^ | -0.1 (-2.1; 1.9) | -2.9 (-6.2; 0.5) | 0.2 (-3.4; 3.9) | 1.4 (-2.3; 5.1) |
| Total cholesterol [mg/dL]^b^ | -0.5 (-2.1; 1.2) | 0.2 (-2.4; 2.9) | -0.4 (-3.6; 3.0) | -0.7 (-3.6; 2.4) |
| hsCRP [mg/dL]^b^ | -6.0 (-12.4; 0.9) | 4.0 (-8.9; 18.7) | -0.9 (-12; 11.7) | **-15.8 (-25.5; -4.8)**** |
| Diastolic blood pressure [mmHg]^a^ | -0.2 (-0.9; 0.6) | 0.3 (-0.9; 1.5) | -0.2 (-1.8; 1.4) | -0.8 (-2.2; 0.6) |
| Systolic blood pressure [mmHg]^a^ | -0.4 (-1.6; 0.8) | 0.5 (-1.3; 2.3) | -1.6 (-3.8; 0.6) | -0.6 (-3.1; 2.0) |
| Fatty liver index^a^ | -0.06 (-0.13; 0.01) | -0.02 (-0.12; 0.08) | 0.01 (-0.13; 0.15) | -0.08 (-0.20; 0.03) |
| **Higher-GI carbohydrates^d^** (energy adjusted) |  |  |  |  |
| Triglycerides [mg/dL]^b^ | 4.2 (-0.8; 9.5) | -0.7 (-7.2; 6.3) | -0.9 (-10.7; 10.0) | **10.8 (0.9; 21.8)*** |
| LDL-cholesterol [mg/dL]^b^ | 0.5 (-2.3; 3.4) | -2.8 (-7.0; 1.6) | 2.4 (-3.3; 8.3) | 1.8 (-3.4; 7.3) |
| HDL-cholesterol [mg/dL]^b^ | **-2.6 (-5.0; -0.2)*** | -0.3 (-4.1; 3.8) | -2.2 (-6.6; 2.3) | **-4.9 (-9.0; -0.6)*** |
| Total cholesterol [mg/dL]^b^ | 0.3 (-1.7; 2.3) | -1.4 (-4.3; 1.7) | 0.1 (-3.9; 4.3) | 1.6 (-2.1; 5.5) |
| hsCRP [mg/dL]^b^ | 3.3 (-5.1; 12.6) | -6.2 (-19.5; 9.2) | -1.2 (-14.8; 14.6) | 13.5 (-2.5; 32.2) |
| Diastolic blood pressure [mmHg]^a^ | 0.2 (-0.8; 1.1) | -0.1 (-1.5; 1.3) | 0.1 (-1.8; 2.1) | 0.3 (-1.4; 2.1) |
| Systolic blood pressure [mmHg]^a^ | 0.0 (-1.4; 1.5) | 0.0 (-2.0; 2.1) | 1.0 (-1.7; 3.7) | -0.3 (-3.4; 2.7) |
| Fatty liver index^a^ | 0.08 (-0.01; 0.17) | 0.01 (-0.12; 0.14) | -0.02 (-0.2; 0.16) | 0.14 (-0.02; 0.29) |
| **Dietary fiber** (energy adjusted) |  |  |  |  |
| Triglycerides [mg/dL]^b^ | -3.3 (-7.6; 1.3) | -2.9 (-8.7; 3.4) | -6.1 (-15.5; 4.4) | 0.8 (-7.4; 9.7) |
| LDL-cholesterol [mg/dL]^b^ | -2.3 (-4.8; 0.3) | -3.4 (-7.3; 0.7) | -2.4 (-7.8; 3.4) | -0.8 (-5.4; 4.0) |
| HDL-cholesterol [mg/dL]^b^ | -0.5 (-2.7; 1.8) | -0.3 (-3.9; 3.5) | -1.0 (-5.5; 3.7) | -0.2 (-4.1; 3.9) |
| Total cholesterol [mg/dL]^b^ | **-2.2 (-3.9; -0.4)*** | -2.7 (-5.4; 0.1) | -1.8 (-5.8; 2.4) | -1.1 (-4.4; 2.2) |
| hsCRP [mg/dL]^b^ | -7.0 (-14.0; 0.6) | -8.7 (-20.8; 5.1) | 3.7 (-10.7; 20.5) | **-12.9 (-24; -0.3)*** |
| Diastolic blood pressure [mmHg]^a^ | -0.3 (-1.1; 0.6) | -0.6 (-1.9; 0.7) | -0.4 (-2.4; 1.6) | -0.4 (-1.9; 1.1) |
| Systolic blood pressure [mmHg]^a^ | -0.4 (-1.8; 0.9) | -0.4 (-2.3; 1.5) | -0.8 (-3.6; 2.0) | -0.4 (-3.2; 2.3) |
| Fatty liver index^a^ | -0.07 (-0.15; 0.01) | -0.02 (-0.13; 0.08) | -0.07 (-0.25; 0.11) | -0.10 (-0.25; 0.05) |
| **Total sugar** (energy adjusted) |  |  |  |  |
| Triglycerides [mg/dL]^b^ | -0.7 (-4.8; 3.6) | -0.7 (-6.7; 5.6) | 1.8 (-5.9; 10.2) | -3.4 (-10.8; 4.7) |
| LDL-cholesterol [mg/dL]^b^ | 0.7 (-1.7; 3.2) | 3.7 (-0.4; 8.0) | -1.2 (-5.4; 3.2) | 1.1 (-3.3; 5.7) |
| HDL-cholesterol [mg/dL]^b^ | -1.9 (-3.9; 0.2) | -3.9 (-7.3; -0.4) | -1.3 (-4.6; 2.2) | -1.1 (-4.7; 2.7) |
| Total cholesterol [mg/dL]^b^ | 0.0 (-1.6; 1.7) | 0.9 (-1.9; 3.7) | -0.1 (-3.2; 3.1) | -0.5 (-3.6; 2.7) |
| hsCRP [mg/dL]^b^ | -2.3 (-9.1; 5.0) | 4.6 (-8.9; 20.2) | 3.1 (-7.9; 15.5) | **-13.0 (-23.4; -1.2)*** |
| Diastolic blood pressure [mmHg]^a^ | -0.1 (-0.9; 0.7) | 0.3 (-1.0; 1.6) | 0.2 (-1.3; 1.7) | -0.8 (-2.2; 0.7) |
| Systolic blood pressure [mmHg]^a^ | -0.2 (-1.4; 1.0) | 0.6 (-1.2; 2.5) | -1.5 (-3.6; 0.6) | -0.2 (-2.8; 2.4) |
| Fatty liver index^a^ | -0.03 (-0.1; 0.04) | -0.01 (-0.11; 0.1) | 0.02 (-0.12; 0.15) | -0.06 (-0.18; 0.06) |

Due to the low absolute number of participants with SIDD and SIRD, these two subtypes were excluded from association and interaction analyses.

Adjusted for age, sex, BMI, current smoking status, total daily energy intake, socioeconomic index, partnership status, physical activity index, total fat intake, total protein intake. Models including serum lipids additionally adjusted for glucose-lowering medication and lipid-lowering medication. Models including hsCRP as dependent variable additionally adjusted for glucose-lowering medication. Models including blood pressure as dependent variable additionally adjusted for antihypertensive medication. Models including fatty liver index as dependent variable additionally adjusted for total daily alcohol intake, glucose-lowering medication and lipid-lowering medication. Dietary GL, low-GI and higher-GI carbohydrates, dietary fiber, total sugar intake, total fat intake, and total protein intake were energy adjusted using the residual method.

Regression coefficients should be interpreted as follows: ^a^ absolute increase of the dependent variable per 1 SD increment in independent variable (Example: A 1 SD increment in dietary GI, i. e. an increase by 3.24, is associated with a decrease in systolic blood pressure by -1.9 mmHg (-5.0; 1.1) among MARD); ^b^ relative increase of the dependent variable per 1 SD increment in independent variable (Example: A 1 SD increment in dietary GL (energy adjusted), i. e. an increase by 22.69, is associated with a decrease in HDL-cholesterol by -4.8% (-9.3; -0.2) among MARD). 1 SD of dietary GI = 3.24; 1 SD of dietary GL (energy adjusted) = 22.69; 1 SD of low-GI carbohydrates (energy adjusted) = 27.04 g; 1 SD of higher-GI carbohydrates (energy adjusted) = 38.67 g; 1 SD of dietary fiber (energy adjusted) = 6.36 g; 1 SD of total sugar (energy adjusted) = 25.20 g.

^c^ Low-GI food sources are defined as GI≤55. ^d^ Higher-GI food sources are defined as GI>55.

**P* <0.05. **Bonferroni corrected *P* <0.0083 (=0.05/m with m=6 for number of parameters of carbohydrate quality).

CI, confidence interval; HDL, high-density lipoprotein; hsCRP, high-sensitivity C-reactive protein; LDL, low-density lipoprotein; MARD, moderate age-related diabetes; MOD, moderate obesity-related diabetes; OAD, oral glucose-lowering drugs; SAID, severe autoimmune diabetes.
